# Infectious Bronchitis Virus as a Vector for the Expression of Heterologous Genes

**DOI:** 10.1371/journal.pone.0067875

**Published:** 2013-06-26

**Authors:** Kirsten Bentley, Maria Armesto, Paul Britton

**Affiliations:** Compton Laboratory, Avian Viral Diseases, The Pirbright Institute, Compton, Newbury, Berkshire, United Kingdom; Kantonal Hospital St. Gallen, Switzerland

## Abstract

The avian coronavirus infectious bronchitis virus (IBV) is the causative agent of the respiratory disease infectious bronchitis of domestic fowl, and is controlled by routine vaccination. To explore the potential use of IBV as a vaccine vector a reverse genetics system was utilised to generate infectious recombinant IBVs (rIBVs) expressing the reporter genes enhanced green fluorescent protein (eGFP) or humanised *Renilla* luciferase (hRluc). Infectious rIBVs were obtained following the replacement of Gene 5 or the intergenic region (IR) with eGFP or hRluc, or the replacement of ORFs 3a and 3b with hRluc. The replacement of Gene 5 with an IBV codon-optimised version of the hRluc gene also resulted in successful rescue of infectious rIBV. Reporter gene expression was confirmed by fluorescence microscopy, or luciferase activity assays, for all successfully rescued rIBVs following infection of primary chick kidney (CK) cells. The genetic stability of rIBVs was analysed by serial passage on CK cells. Recombinant IBV stability varied depending on the genome region being replaced, with the reporter genes maintained up to at least passage 8 (P8) following replacement of Gene 5, P7 for replacement of the IR and P5 for replacement of ORFs 3a and 3b. Codon-optimisation of the hRluc gene, when replacing Gene 5, resulted in an increase in genome stability, with hRluc expression stable up to P10 compared to P8 for standard hRluc. Repeated passaging of rIBVs expressing hRluc at an MOI of 0.01 demonstrated an increase in stability, with hRluc expression stable up to at least P12 following the replacement of Gene 5. This study has demonstrated that heterologous genes can be incorporated into, and expressed from a range of IBV genome locations and that replacement of accessory Gene 5 offers a promising target for realising the potential of IBV as a vaccine vector for other avian pathogens.

## Introduction

Coronaviruses are positive-sense RNA viruses with large genomes ranging in size from approximately 26 to 31 kb, and are known to infect a wide range of mammalian and avian species, with species and tissue specific tropisms. All coronaviruses share a similar genome organisation with Gene 1, the replicase gene, located at the 5′ end of the genome and the structural and group-specific accessory genes clustered at the 3′ end. A process of discontinuous transcription during negative strand synthesis, regulated by short AU rich sequences known as transcription regulatory sequences (TRSs), leads to expression of the structural and accessory proteins as a nested set of subgenomic (sg) mRNAs (reviewed in [1]–[3]). The avian gammacoronavirus IBV is a highly infectious pathogen of domestic fowl that causes disease in chickens of all ages and despite vaccination, using both live attenuated and inactivated vaccines, is responsible for major economic losses to poultry industries worldwide as a result of poor weight gain and decreased egg production [4]–[9].

The large size of coronavirus genomes, combined with the possibility of expressing heterologous genes via the generation of novel sg mRNAs, has meant that coronaviruses have long been attractive targets for use as viral-vector vaccines and gene delivery systems. Previous work by ourselves and others has shown that heterologous genes can be expressed utilising TRSs from coronavirus defective RNAs (D-RNAs) in the presence of helper virus [10]–[14]. In recent years a number of reverse genetics systems have been successfully developed to produce full-length cDNAs from several coronaviruses including TGEV, human coronavirus 229E, SARS-CoV and human coronavirus NL63 [15]–[19]; with these advances making it possible to investigate the potential of using coronaviruses as vaccine vectors. A reverse genetics system for IBV, utilizing vaccinia virus, has also been established and so made it possible to explore the use of rIBVs for vaccine development [20]–[22].

To date a number of studies have demonstrated the generation of infectious recombinant coronaviruses that are able to express heterologous genes, a key requirement of any vaccine vector [23]–[27]. These studies, as outlined by de Haan *et al*. [28], [29], showed that the expression of heterologous proteins may be dependent on a number of factors, including the genetic background of the virus, the spatial location in the virus genome at which the heterologous gene is inserted and, if replacing genes, how essential these genes are to the replication of the coronavirus. Due to the nature of coronavirus sg mRNA transcription the expression of heterologous genes may be achieved by expression from an existing sg mRNA either via insertion of a gene cassette or by replacement of existing genes. Alternatively, heterologous genes can be expressed from an additional, newly synthesised sg mRNA as demonstrated from IBV D-RNAs [12], [14] or TGEV minigenomes [30], although these were often found to be genetically unstable. Recombinant viruses consisting of full-length virus genomes showed that stability was often greatest following the replacement of the non-essential accessory genes [24], [27], [29], [31], while alterations to coronavirus structural genes appeared to be less well tolerated with reduced virus stability, or an inability to rescue infectious virus [24], [32].

Given the potential for coronaviruses as vaccine vectors, and the continual requirement for IBV vaccination, IBV has the potential to be a suitable vaccine vector for the generation of bivalent vaccines that protect against not only IBV, but also additional avian diseases as determined by the expression of a heterologous gene. Other avian viruses such as the paramyxovirus Newcastle disease virus, and the avipoxvirus fowlpox virus have previously been evaluated for use as vectors, not only as vaccines for veterinary use [33], [34] but also for vaccine and gene therapy use in humans [35]–[37] and reviewed in [38], [39] demonstrating the potential for using avian viruses as vectors. Additionally, IBV possesses two accessory genes, Gene 3 and Gene 5, and the recently characterised IR, all of which have been shown to be dispensable for virus replication in cell culture [27], [40]–[42] and therefore provide potential targets for replacement with a heterologous gene.

In this study we used our IBV reverse genetics system [20], [21], based on the avirulent Beaudette strain of IBV, to generate rIBVs in which different regions of the IBV genome were replaced with the reporter genes eGFP or hRluc. The resulting rIBVs were assessed to investigate whether: (1) the size and type, (2) the spatial position or (3) the nucleotide sequence of the gene introduced were important factors in determining the possibility for utilising IBV as a vaccine vector. Reporter genes were inserted under the control of existing TRSs in the case of the replacement of Gene 3 and Gene 5, or under the control of an additionally inserted copy of an IBV canonical consensus TRS in the case of the replacement of the IR. Successfully rescued viruses were characterised and subsequently investigated for genetic stability. The effect of codon-optimisation of the heterologous gene to be more ‘IBV-like’ in nucleotide sequence was also investigated. Our results demonstrated that heterologous gene expression could be achieved in cell culture for all successfully rescued rIBVs, although genome stability varied depending on the genomic location of the reporter gene and the MOI at which rIBVs were passaged *in vitro*. The results of this study suggest that the replacement of Gene 5 with heterologous genes represents a promising target for the generation of IBV-based vaccine vectors.

## Materials and Methods

### Ethics Statement

Primary CK cells were prepared by The Pirbright Institute cell culture department from chickens produced in the Institute's poultry production unit. The chickens were sacrificed by trained staff under a schedule 1 procedure, cervical neck dislocation, a procedure that does not fall under any UK Home Office licence requirements as no procedures were carried out on live animals and therefore did not fall under any local or EU ethical requirements. The kidneys were removed from the sacrificed chickens for preparation of the primary CK cell cultures. The work was done in a designated establishment, The Pirbright Institute, Compton Laboratory.

### Cells and Viruses

Primary chicken kidney (CK) cells were prepared from 2-3 week old specific pathogen free (SPF) Rhode Island Red chickens as described previously [43]. All IBVs were propagated and titrated on primary CK cells using BES (N, N-Bis(2-hydroxyethyl)-2-aminoethanesulphonic acid) cell maintenance medium [44].

### Construction of Modified IBV cDNA Plasmids and Generation of Recombinant Viruses

All recombinant viruses were generated with a genomic backbone of Beau-R and all nucleotide numbers refer to genome positions in this virus (GenBank Number AJ311317). Reporter genes eGFP and hRluc were amplified from plasmids pEGFP-C1 (Clontech) and pGL4.75hRluc/CMV (Promega), respectively. Modified fragments of IBV cDNA for the replacement of Gene 5 by eGFP or hRluc, between nucleotides 25479 and 25702 inclusively, were generated by overlapping PCR. Three fragments were amplified with primer pairs: (1) ^24979^M – ^25487^IR (^24979^M: 5′-TTT***GGCGCGCC***GCTAAGTGTGAACCAGACCACTTGCC-3′ and ^25487^IR-eGFP: 5′-GCTCCTCGCCCTTGCTCACCATCGTCCGTATTTGTTAAGTTTTTG-3′ or ^25487^IR-hRluc: 5′- GTCGTACACCTTGGAAGCCATCGTCCGTATTTGTTAAGTTTTG-3′), (2) eGFP or hRluc (eGFP-F: 5′-CAAAAACTTAACAAATACGGACGATGGTGAGCAAGGGCGAGGAGC-3′ and eGFP-R: 5′-GCTATTGCTCCGCGAAAAGGTTATTACTTGTACAGCTCGTCC-3′) or (hRluc-F: CAAAAACTTAACAAATACGGACGATGGCTTCCAAGGTGTACGAC-3′ and hRluc-R: 5′- GCTATTGCTCCGCGAAAAGGTTATTACTGCTCGTTCTTCAGC-3′) and (3) ^25702^5b – ^26250^N (^25702^5b-eGFP: 5′- GGACGAGCTGTACAAGTAATAACCTTTTCGCGGAGCAATAGC-3′ or ^25702^5b-hRluc: 5′- GCTGAAGAACGAGCAGTAATAACCTTTTCGCGGAGCAATAGC-3′ and ^26250^N: 5′- CCC***TTAATTAA***GTCGATCTAGATTTAGTATCAGCACCC-3′). Underlined nucleotides are derived from reporter gene sequences and bold italics show inserted restriction sites *Asc*I and *Pac*I. Fragments were joined by PCR, and cloned into *Asc*I/*Pac*I digested pGPTNEB193 as described previously [20], [42], [44]–[46] to generate plasmids pGPT-eGFPΔ5ab and pGPT-hRlucΔ5ab.

To create altered nucleotide gene sequences for IBV codon-optimised eGFP (IBVeGFP) and hRluc (IBVhRluc) a codon usage table was generated from IBV coding sequences (Table 1). Wild type amino acid sequences for eGFP (pEGFP-C1) and hRluc (pGL4.75hRluc/CMV) were back translated utilizing the alternative IBV preferred codons, as per Table 1, to generate IBV codon-optimised reporter gene sequences which were subsequently synthesised within the context of the IBV cDNA fragment described above by Geneart® (Life Technologies). Only the synthetic sequence representing IBVhRluc could be cloned into pGPTNEB193 by Geneart® to give plasmid pGPT-IBVhRlucΔ5ab.

**Table 1:** IBV Codon Usage.

Codon Frequency per thousand (Number)*			
UUU 42.3 (12136)	UCU 22.2 (6359	UAU 29.9 (8580)	UGU 22.3 (6395)
UUC 10.0 (2859)	UCC 3.1 (879)	UAC 12.2 (3487)	UGC 5.8 (1653)
UUA 18.6 (5339)	UCA 18.3 (5254)	UAA 1.3 (376)	UGA 1.0 (292)
UUG 14.3 (4101)	UCG 3.0 (871)	UAG 0.4 (113)	UGG 14.8 (4247)
CUU 24.9 (7142)	CCU 18.5(5312)	CAU 8.7 (2500)	CGU 12.2 (3501)
CUC 5.4 (1541)	CCC 5.0(1428)	CAC 5.3 (1533)	CGC 5.6 (1612)
CUA 11.4 (3266)	CCA 20.0 (5733)	CAA 24.2 (6937)	CGA 2.5 (708)
CUG 7.2 (2057)	CCG 4.0 (1138)	CAG 18.8 (5381)	CGG 1.5 (424)
AUU 27.2 (7813)	ACU 26.5 (7618)	AAU 44.0 (12624)	AGU 21.6 (6186)
AUC 5.6 (1596)	ACC 4.8 (1379)	AAC 12.8 (3671)	AGC 5.9 (1690)
AUA 19.7 (5643)	ACA 20.9 (6004)	AAA 31.1 (8924)	AGA 16.0 (4605)
AUG 15.5 (4434)	ACG 4.9 (1415)	AAG 30.6 (8769)	AGG 8.2 (2341)
GUU 33.3 (9556)	GCU 25.7 (7371)	GAU 38.3 (10980)	GGU 39.4 (11297)
GUC 7.7 (2198)	GCC 8.0 (2302)	GAC 15.6 (4482)	GGC 8.1 (2332)
GUA 18.8 (5383)	GCA 27.4 (7874)	GAA 26.2 (7506)	GGA 18.6 (5347)
GUG 14.6 (4199)	GCG 6.7 (1916)	GAG 17.4 (4979)	GGG 4.7 (1353)

* IBV codon usage was derived from 785 coding sequences (286941 codons).

For the replacement of the IR by eGFP or hRluc between nucleotides 25192 and 25459 reporter gene sequences were amplified by PCR to add 5′-*Nhe*I and 3′-*Xma*I restriction sites along with a copy of the Gene 5 TRS at the 5′ end: (eGFP-F: 5′- TTT***GCTAGC***GGCCAACTTAACAAATACGGACGATGGTGAGCAAGGGCGAGG and eGFP-R: 5′- AAA***CCCGGG*** TTATTACTTGTACAGCTCGTCCATGCC-3′) or (hRluc-F: 5′- TTT***GCTAGC***GGCCAACTTAACAAATACGGACGATGGCTTCCAAGGTGTACG-3′ and hRluc-R: 5′- AAA***CCCGGG*** TTATTACTGCTCGTTCTTCAGCACGCGC-3′). Underlined nucleotides are derived from reporter gene sequences and bold italics show inserted restriction sites *Nhe*I and *Xma*I. Sequences were cloned into *Nhe*I/*Xma*I digested pGPTΔIR as described previously [42] to give plasmids pGPT-eGFPΔIR and pGPT-hRlucΔIR.

For the replacement of ORFs 3a and 3b between nucleotides 23857 and 24203 inclusively modified IBV cDNA fragments were synthesised and cloned into pGPTNEB193 by Geneart® to give plasmids pGPT-eGFPΔ3ab and pGPT-hRlucΔ3ab. A single copy of the duplicated Gene 5 TRS and flanking nucleotides (5′-GUUUUACUUAACAAAUACGGACG-3′) was inserted between the reporter gene termination codon and the ORF 3c initiation codon beginning at nucleotide 24204 for transcription of ORF 3c sg mRNA.

The control rIBV BeauRΔ5ab was generated by deletion of the IBV cDNA sequence, nucleotides 25451–25693, covering the gene 5 TRS, ORF 5a and the N-terminal end of ORF 5b from pGPT-K5ScAUG5b [40]. Essentially the introduced *Kpn*I site was treated with T4 DNA polymerase and ligated to the introduced *Sca*I site downstream of the ORF5b AUG resulting in plasmid pGPT-Δ5ab.

Modified regions of the IBV cDNA within plasmids pGPT-Δ5ab, pGPT-eGFPΔ5ab, pGPT-hRlucΔ5ab, pGPT-IBVhRlucΔ5ab, pGPT-eGFPΔIR, pGPT-hRlucΔIR, pGPT-eGFPΔ3ab and pGPT-hRlucΔ3ab were introduced into the IBV Beaudette full-length cDNA within the vaccinia virus genome by homologous recombination using transient dominant selection as described previously [20], [44], [47]. Infectious rIBVs were recovered from DNA isolated from the recombinant vaccinia viruses containing the correctly modified IBV cDNAs and passaged four times on CK cells prior to experimental use [20], [44], [47].

### RNA extraction and RT-PCR

Viral RNA was extracted from cell supernatants using the RNeasy Mini Kit (Qiagen) following the manufacturer's instruction for the RNA clean up protocol. cDNA was synthesised using SuperScript III (Life Technologies) and a random primer as per manufacturer's instructions. cDNA was amplified by PCR using primer pairs that flanked the inserted reporter gene: for Δ5ab recombinant viruses (5′-GAGCTATTAACGGTGTTACC-3′) and (5′-AATCTAATCCTTCTCTCAGA-3′), for ΔIR recombinant viruses (5′-GAATGGTGTTCTTTATTG-3′) and (5′-TCTAACACTCTAAGTTGAG-3′), and for Δ3ab recombinants (5′-TGACGAATTGTCAAAATG-3′) and (5′-AGACAGACACGCAAACACTG-3′). PCR cycling parameters were as follows: 1 cycle of 94°C for 2 min; 25 cycles of 94°C for 30 sec, 50°C for 30 sec, 72°C for 30 sec; and a final extension step of 72°C for 2 min.

### Growth Kinetics of rIBVs

Individual wells of six-well plates of confluent CK cells were infected with 1×10^5^ pfu of virus and incubated for 1 h at 37°C, 5% CO_2_ after which cells were washed twice with PBS and 2 ml of fresh 1×BES media added. Extracellular virus was harvested at 1, 8, 12, 24, 48 and 72 hours post infection (hpi) and subsequently analysed in triplicate by plaque assay on CK cells for progeny virus.

### Luciferase Assays

Cell lysates were prepared from CK cells infected with recombinant IBVs expressing hRluc gene and luciferase activity measured using the *Renilla* Luciferase Assay System (Promega) as per manufacturer's instructions. Relative light unit (RLU) values were obtained using a GloMax® 20/20 luminometer (Promega) with integration over 10-seconds with a 2-second delay.

### Northern Blot Analysis

Total RNA was extracted from CK cells 24 hpi using the RNeasy Mini Kit (Qiagen) and mRNA purified using the Poly(A)Purist™ MAG Kit (Ambion) according to manufacturer's instructions. Northern blot analysis was carried out with the NorthernMax®-Gly Kit (Ambion) as described previously [42]. Briefly, viral mRNA transcripts were denatured at 50°C for 30 min in glyoxal load dye followed by separation on a 0.8% LE-agarose gel. RNA was transferred to BrightStar®-Plus positively charged nylon membrane (Ambion) using capillary action for 2 h, cross-linked by treatment with UV light using the auto-crosslink function on a Stratalinker UV Crosslinker (Stratagene) and prehybridised for 30 min with ULTRAhyb® buffer at 42°C. Blots were probed with a DNA probe specific to the 3′ end of IBV and hybridised overnight at 42°C followed by washing and development with the BrightStar® BioDetect™ Kit.

### Western Blot Analysis

Confluent monolayers of CK cells were infected with 1×10^5^ pfu virus. At 20 hpi cells were washed twice with PBS, lysed in buffer containing 20 mM Tris, 150 mM NaCl, 5 mM EDTA, 0.3% Triton X100 and 1X protease inhibitor cocktail (Sigma) and clarified by centrifugation. Samples were denatured in NuPAGE® LDS Sample Buffer (4X) (Life Technologies), heated for 10 min at 70°C, separated by polyacrylamide gel electrophoreses (PAGE) using NuPAGE® 10% Bis-Tris pre-cast polyacrylamide gels (Life Technologies) and transferred to nitrocellulose membrane (Amersham). Separated proteins were probed with polyclonal rabbit sera against IBV diluted 1∶2500 and a polyclonal antibody against beta-actin diluted 1∶500 (Abcam). Enhanced GFP expression was confirmed using a polyclonal antibody against GFP diluted 1∶1000 (Abcam) and *Renilla* luciferase expression was confirmed using a polyclonal antibody against *Renilla* luciferase diluted 1∶1000 (MBL International). Bound antibody was visualized using an enhanced-chemiluminescence detection system (ECL) (Millipore).

## Results

### Design of recombinant IBVs

**Figure 1:**
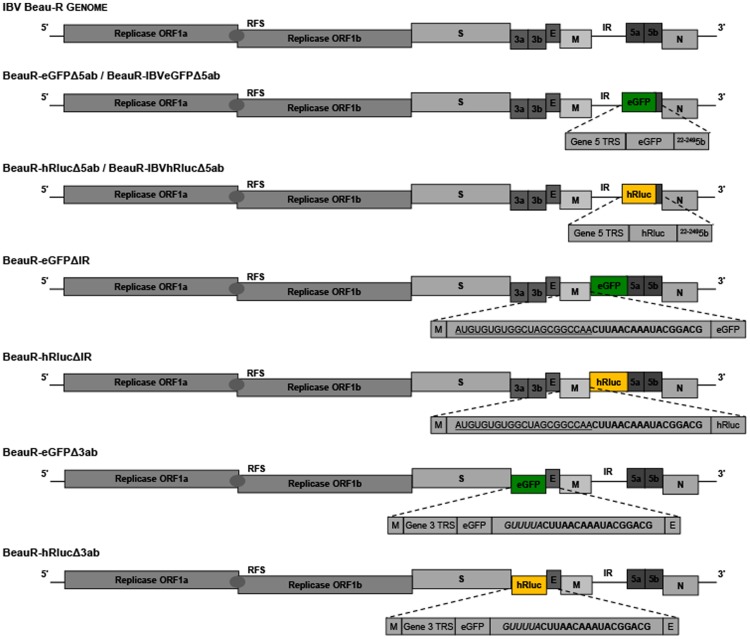
Design of rIBV constructs. Schematic of wild type Beau-R and rIBV genomes displaying position of reporter genes; with eGFP shown as green boxes and hRluc shown as yellow boxes. As the schematic diagrams of the rIBVs containing codon optimised versions, IBVeGFP and IBVhRluc, of the eGFP and hRluc genes in place of Gene 5 were the same as the normal reporter genes separate diagrams are not included. Expanded regions show the nature of the TRS controlling expression of each reporter gene with use of existing sequences referred to as Gene 5 TRS or Gene 3 TRS. For Δ5ab rIBVs the nucleotides remaining for 5b are given. For ΔIR rIBVs underlined sequence relates to residual IR sequence and cloning sites required for construction of reverse genetics selection vector. In bold is shown the Gene 5 8-nucleotide TRS plus nine 3′-flanking nucleotides. In addition for Δ3ab rIBVs the six 5′-flanking nucleotides from the Gene 5 TRS are shown in italics.

In order to investigate the potential of IBV as a vaccine vector we first designed a series of rIBVs that could be used to assess the ability of IBV to express heterologous proteins and subsequently determine the stability of each rIBV. Based on the Beau-R molecular clone of the Beaudette-CK strain of IBV [21], we designed eight rIBVs in which three different IBV genome regions, Gene 5, ORFs 3a and 3b or the IR, were replaced with the reporter genes eGFP or hRluc, or codon-optimised reporter genes IBVeGFP and IBVhRluc (Figure 1). The growth characteristics of the rIBVs were compared to the control viruses BeauRΔ5ab (this paper), BeauRΔ3ab [41] and BeauRΔIR [42] in which the regions being replaced were deleted. In the design of each construct measures were taken to ensure that the transcription of other IBV genes was not affected by the loss of virus sequences or the incorporation of the reporter gene sequences. Recombinant IBVs, BeauR-eGFPΔ5ab and BeauR-hRlucΔ5ab, consisted of direct replacement of IBV Gene 5 sequence, beginning at the ORF 5a initiation codon, and ending at amino acid seven of ORF 5b in order to maintain the genomic sequence upstream of the N gene TRS. Expression of eGFP or hRluc from BeauR-eGFPΔ5ab and BeauR-hRlucΔ5ab was achieved via the existing Beaudette Gene 5 TRS.

Recombinant IBVs, BeauR-eGFPΔ3ab and BeauR-hRlucΔ3ab, consisted of the direct replacement of ORF 3ab sequence, beginning at the second nucleotide (U) of the ORF 3a initiation codon in order to preserve the overlapping spike termination codon. A stretch of 20 nucleotides, corresponding to the 3′ end of ORF 3b, was maintained to avoid disruption of ORF 3c, encoding the envelope (E) protein. The introduced reporter genes, replacing ORFs 3ab, were expressed utilising the existing Gene 3 TRS. To ensure expression of the E protein the insertion of an artificial TRS was required directly downstream of the reporter gene sequence for the transcription of a sg mRNA for translation of the E protein. As the full extent of the sequences required for efficient sg mRNA transcription are currently unknown for IBV we designed a TRS containing a copy of the consensus IBV TRS with flanking nucleotides derived from the Gene 5 TRS (5′-GUUUUACUUAACAAAUACGGACG-3′) based on our knowledge of the expression of foreign genes from IBV D-RNAs [12], [14]. Inclusion of six 5′-flanking, and nine 3′-flanking nucleotides was based on an extension of previous findings demonstrating that the four nucleotides flanking either side of the consensus sequence were important for efficient sg mRNA expression, and that these nucleotides would increase complementarity with the leader sequence as presumably is the case for Gene 5 expression [48].

Recombinant IBVs, BeauR-eGFPΔIR and BeauR-hRlucΔIR, were designed to express reporter genes from a novel sg mRNA. The Beaudette IR sequence was replaced, between nucleotides 25192 and 25459 of the Beau-R genome, with a reporter gene construct in which a copy of the consensus IBV TRS with 3′ flanking nucleotides derived from the Gene 5 TRS (5′-CUUAACAAAUACGGACG-3′), was placed upstream of the reporter gene sequence for the transcription of an additional sg mRNA for translation of eGFP or hRluc. As with the inserted TRS for expression of E from BeauR-hRlucΔ3ab 3′-flanking nucleotides were added to increase complementarity to the leader sequence and improve chances of sg mRNA synthesis. In this case no 5′-flanking nucleotides were added, as due to the cloning procedure required for these rIBVs additional sequences would be generated 5′ to the TRS that it was hoped would act to position to TRS correctly for sg mRNA synthesis and provide additional, although limited, sequence complementarity.

**Figure 2:**
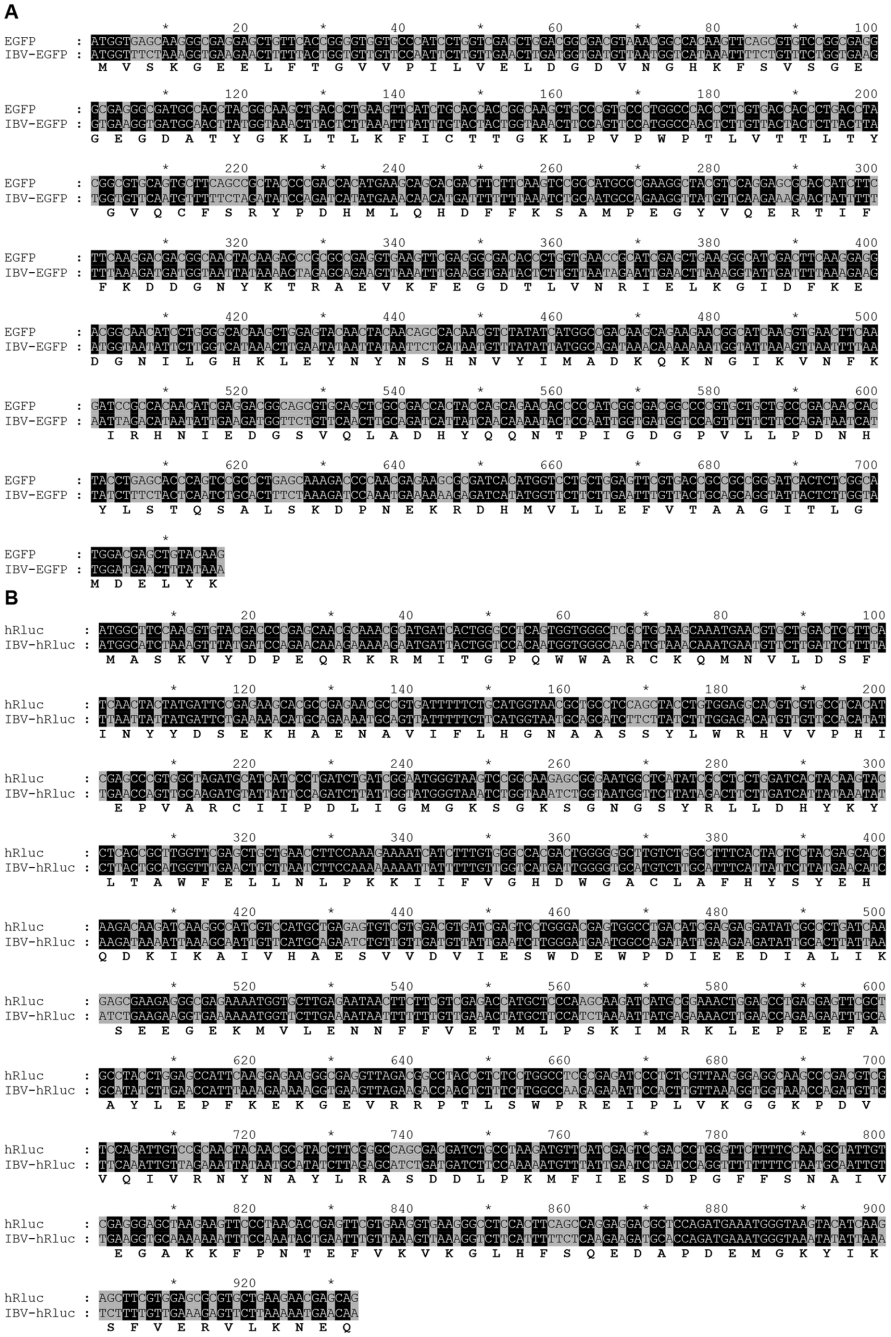
Codon-optimised reporter gene sequences. Wild type reporter gene sequences were back translated using the IBV preferred codon, as indicated in Table 1, to generate a new nucleotide sequence codon-optimised for IBV. Wild type nucleotide sequences for (A) eGFP and (B) hRluc were aligned to codon-optimised sequences IBVeGFP and IBVhRluc, respectively. Nucleotides highlighted in black remained the same and those that modified to give IBV-optimised codons highlighted in grey, the amino acid sequences are shown below the alignments.

In addition to genome position, we also investigated the effect of codon-optimisation of the heterologous gene by redesigning the gene sequence so that codon usage maximally represented that of IBV. Using a codon usage table (details at www.kazusa.or.jp/codon/) (Table 1) and the Emboss backtransseq programme (details at http://emboss.open-bio.org/) new eGFP and hRluc nucleotide sequences, IBVeGFP and IBVhRluc, were generated and synthesised with 34 and 29% of nucleotides changed, respectively (Figure 2). The final two rIBVs, BeauR-IBVeGFPΔ5ab and BeauR-IBVhRlucΔ5ab, were subsequently designed in which Gene 5 was replaced, as above, with the synthesised codon-optimised reporter genes.

### Characterisation of rIBVs expressing eGFP or hRluc following replacement of Gene 5

**Figure 3:**
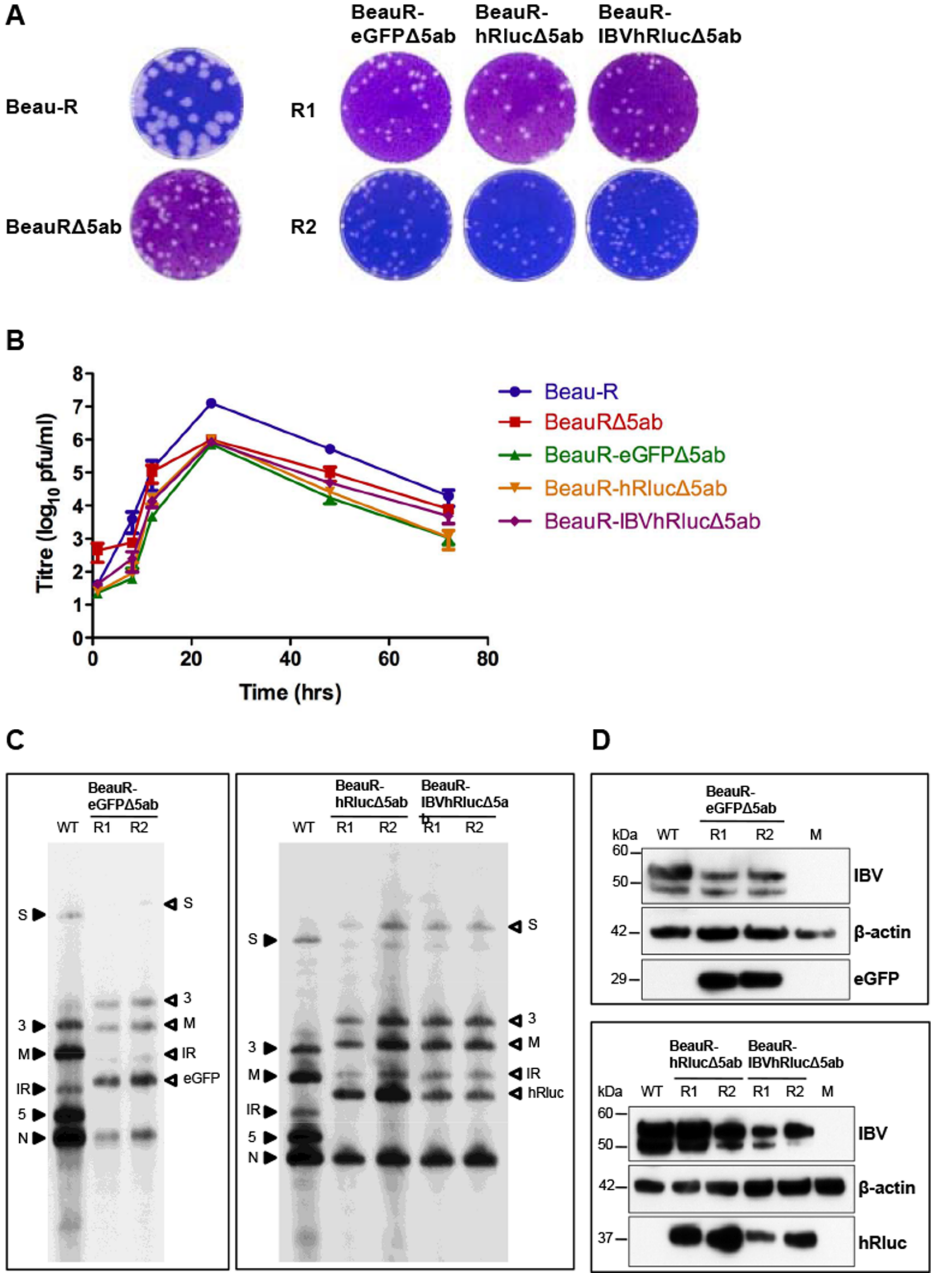
Characterisation of rIBVs with replacement of Gene 5 with eGFP or hRluc. (A) Monolayers of CK cells were infected with wild type virus (Beau-R), control virus (BeauRΔ5ab) or P4 of rIBVs BeauR-eGFPΔ5ab, BeauR-hRlucΔ5ab or BeauR-IBVhRlucΔ5ab and cytopathic effect analysed by plaque assay. (B) Growth kinetics of Beau-R, BeauRΔ5ab and rIBVs in CK cells. Extracellular virus was harvested at various times post infection and the progeny virus titres determined by plaque assay. Error bars represent standard deviation (SD) of results from three experiments assayed in triplicate. (C) Northern blot analysis of purified mRNA extracted from CK cells infected with 1×10^5^ pfu wild type, parental virus, Beau-R (WT) and replicates R1 and R2 of P4 BeauR-eGFPΔ5ab (left panel) or BeauR-hRlucΔ5ab and BeauR-IBVhRlucΔ5ab (right panel). Purified mRNA was not quantified prior to loading on gel and is shown only to demonstrate that sg mRNAs were generated as expected. RNAs were hybridised with a DNA probe specific to the IBV 3′ UTR. Closed arrowheads indicate the sizes of sg mRNAs from Beau-R and open arrowheads indicating the altered sizes of the sg mRNAs from the rIBVs. (D) Western blot analysis of reporter gene expression. Proteins in cell lysates of CK cells infected with wild type, parental virus, Beau-R (WT) or P4 BeauR-eGFPΔ5ab (top panel) or BeauR-hRlucΔ5ab and BeauR-IBVhRlucΔ5ab (lower panel) were analysed by western blot analysis using anti-IBV, anti-eGFP or anti-hRluc and anti-actin antibodies. Cell lysates from uninfected cells (M) were included as a negative control.

Utilising our IBV reverse genetics system [20], [21] we rescued two replicates, R1 and R2, of rIBVs BeauR-eGFPΔ5ab, BeauR-hRlucΔ5ab and BeauR-IBVhRlucΔ5ab, in which Gene 5 was replaced with reporter genes eGFP, hRluc or IBVhRluc, respectively. A potential rIBV, BeauR-IBVeGFPΔ5ab containing the codon-optimised eGFP gene, was not possible, as we were unable to insert a copy of the IBV codon-optimised eGFP sequence into the selection plasmid required for modifying the IBV cDNA in our reverse genetics system. Each rescued rIBV was subsequently passaged to P4 on CK cells for further analysis. The successful rescue of infectious rIBVs was confirmed by formation of IBV-induced plaques on CK cells (Figure 3A), although it was observed that plaques for all Δ5ab rIBVs were approximately 2 mm smaller in diameter than plaques observed for wild type Beau-R.

To analyse the effects of deleting IBV Gene 5, and subsequent insertion of the reporter genes in place of Gene 5, the replication kinetics of R1 isolates of BeauR-eGFPΔ5ab, BeauR-hRlucΔ5ab and BeauR-IBVhRlucΔ5ab were compared to wild type Beau-R and control BeauRΔ5ab viruses (Figure 3B). Similar replication kinetics were observed for all three rIBVs and for control virus BeauRΔ5ab. However, peak titres at 24 hpi for all the rIBVs lacking the Gene 5 proteins were approximately 1-log_10_ lower, at 1×10^6^ pfu/ml, than wild type Beau-R. Additionally, a 2-log_10_ increase in titre was noted for Beau-R compared to a<0.5-log_10_ increase at 8 hpi for all rIBVs suggesting a delay in replication or release of the rIBVs lacking Gene 5. Combined with the observation of smaller plaque sizes these results suggested that the replacement or deletion of Gene 5 had a minor detrimental effect on virus replication in CK cells. Northern blot analysis of rIBVs sg mRNAs demonstrated an increase in the size of the sg mRNA initiated from the Gene 5 TRS when compared to parental virus Beau-R, and which corresponded to the presence of eGFP or hRluc sequences (Figure 3C). Corresponding expected increases in size, due to the presence of the reporter gene sequences, were also observed for all the larger sg mRNAs synthesised upstream of the Gene 5 replacement site, due to the nested nature of coronavirus sg mRNAs.

**Figure 4:**
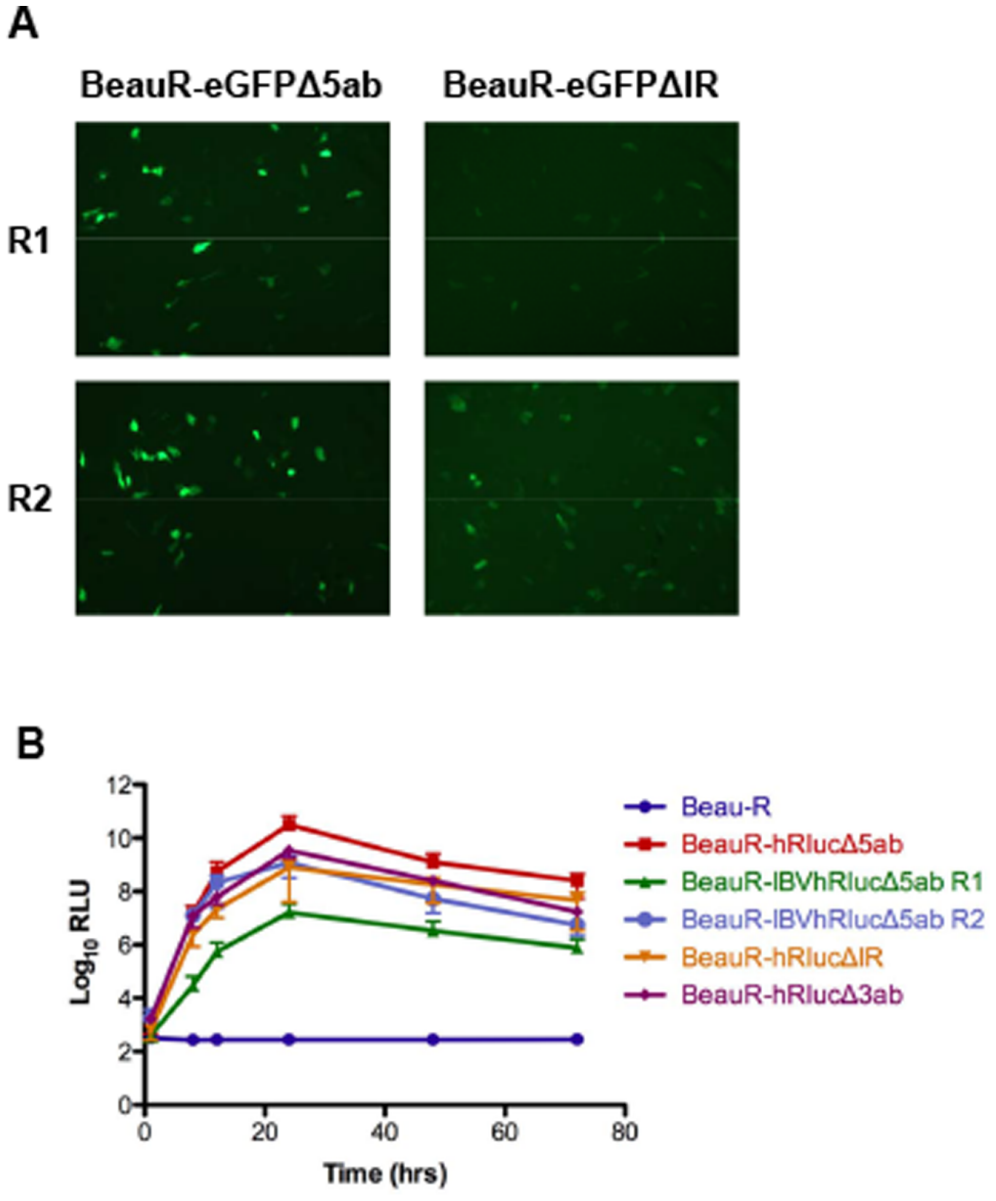
Analysis of reporter gene expression. (A) Expression of eGFP observed by fluorescence microscopy in CK cells infected with R1 and R2 of BeauR-eGFPΔ5ab and BeauR-eGFPΔIR. (B) hRluc expression, as relative light units (RLU), was detected in CK cells infected with R1 of BeauR-hRlucΔ5ab, BeauR-hRlucΔIR, BeauR-hRlucΔ3ab and with R1 and R2 of BeauR-IBVhRlucΔ5ab. Error bars represent SD of results from three experiments assayed in triplicate.

**Figure 5:**
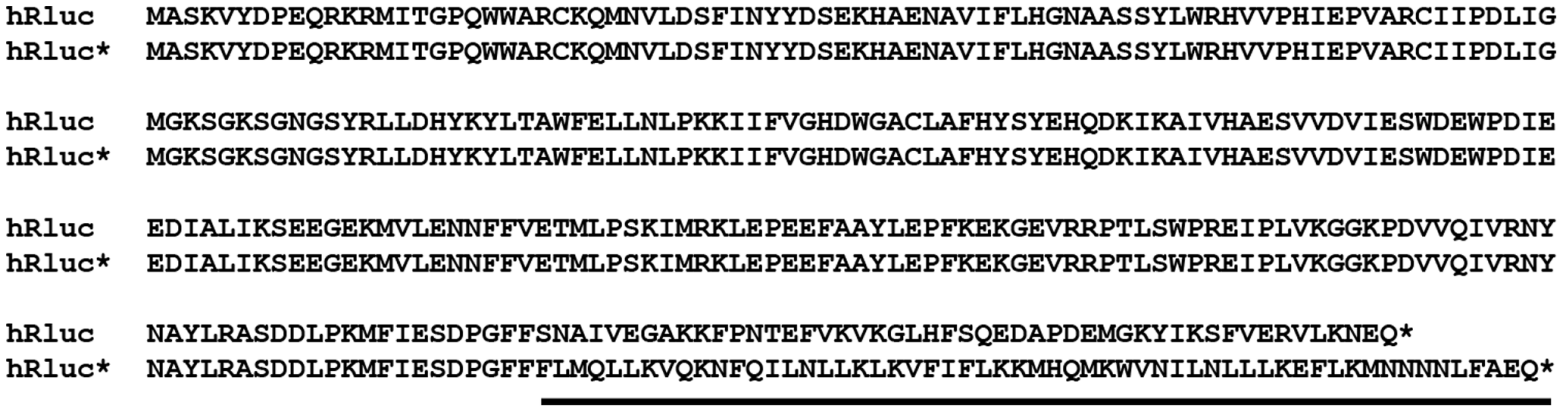
Amino acid alignment of mutated hRluc gene in the R1 of BeauR-IBVhRlucΔ5ab. The amino acid sequence of the mutated hRluc gene (hRluc*) identified in R1 of BeauR-IBVhRlucΔ5ab is shown aligned to the hRluc gene sequence. The modified region in hRluc* sequence is underlined.

Heterologous protein expression was confirmed for the three rIBVs by western blot analysis of rIBV-infected CK cell lysates using anti-GFP and anti-Renilla luciferase antibodies (Figure 3D). In addition, GFP-induced fluorescence was observed in CK cells infected with P4 of rIBV BeauR-eGFPΔ5ab (Figure 4A). To confirm the expression of active *Renilla* luciferase from rIBVs BeauR-hRlucΔ5ab and BeauR-IBVhRlucΔ5ab, luciferase activity was measured in CK lysates during time course assays for replication kinetics, and was found to correlate with levels of virus titre during infection (Figure 4B). When compared to BeauR-hRlucΔ5ab, and subsequently R2 of BeauR-IBVhRlucΔ5ab, the luciferase activity of BeauR-IBVhRlucΔ5ab R1 reached approximately 2-log_10_ lower peak levels at 24 hpi. Sequence analysis of P4 of BeauR-IBVhRlucΔ5ab R1 established that a mutation had arisen in the codon-optimised hRluc gene during initial passage of rescued virus on CK cells. The mutation, an insertion of two uracil residues at nucleotide position 787 within the codon-optimised hRluc gene, led to a frameshift and alteration of the hRluc amino acid sequence downstream of amino acid 262. This frameshift resulted in a modified IBVhRluc protein, eight amino acids longer than the wild type codon-optimised version (Figure 5). Analysis of BeauR-IBVhRlucΔ5ab R2 demonstrated luciferase activity levels more closely mirroring those of BeauR-hRlucΔ5ab, suggesting the mutation within the hRluc gene of BeauR-IBVhRlucΔ5ab R1 was the cause of the observed reduction in luciferase activity (Figure 4B).

### Characterisation of rIBVs expressing eGFP or hRluc following replacement of the Beaudette IR

**Figure 6:**
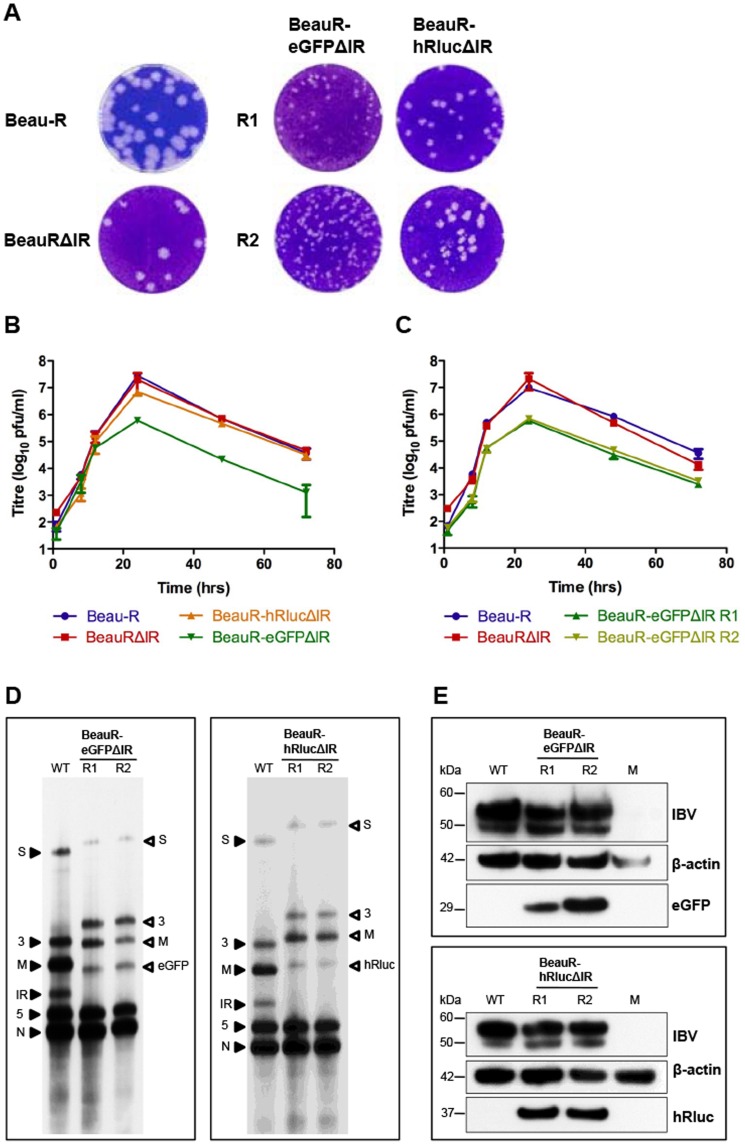
Characterisation of rIBVs with replacement of the IR with eGFP or hRluc. (A) Monolayers of CK cells were infected with wild type virus (Beau-R), control virus (BeauRΔIR) or P4 of rIBVs BeauR-eGFPΔIR or BeauR-hRlucΔIR and cytopathic effect analysed by plaque assay. (B) Growth kinetics of Beau-R, BeauRΔIR and R1 of BeauR-eGFPΔIR and BeauR-hRlucΔIR in CK cells. Extracellular virus was harvested at various times post infection and the progeny virus titres determined by plaque assay. Error bars represent SD of results from three experiments assayed in triplicate. (C) Comparison of growth kinetics of Beau-R, BeauRΔIR and R1 and R2 of BeauR-eGFPΔIR in CK cells. Extracellular virus was harvested at various times post infection and the progeny virus titres determined by plaque assay. Error bars represent SD of results from three experiments assayed in triplicate. (D) Northern blot analysis of purified mRNA extracted from CK cells infected with 1×10^5^ pfu wild type, parental virus, Beau-R (WT) and replicates R1 and R2 of P4 BeauR-eGFPΔIR (left panel) or BeauR-hRlucΔIR (right panel). Purified mRNA was not quantified prior to loading on gel and is shown only to demonstrate that sg mRNAs were generated as expected. RNAs were hybridised with a DNA probe specific to the IBV 3′ UTR. Closed arrowheads indicate the sizes of sg mRNAs from Beau-R and open arrowheads indicating the altered sizes for the sg mRNAs produced from the rIBVs. (E) Western blot analysis of reporter gene expression. Proteins in cell lysates of CK cells infected with wild type, parental virus, Beau-R (WT) or P4 BeauR-eGFPΔIR (top panel) or BeauR-hRlucΔIR (lower panel) were analysed by western blot analysis using anti-IBV, anti-eGFP or anti-hRluc and anti-actin antibodies. Cell lysates from uninfected cells (M) were included as a negative control.

As with the replacement of Gene 5 we used our IBV reverse genetics system to generate two replicates each of rIBVs BeauR-eGFPΔIR and BeauR-hRlucΔIR, in which the heterologous gene sequences replaced the Beaudette IR sequence between the end of the M gene and upstream of the Gene 5 TRS. We have recently shown that the IBV IR is produced as a novel sg mRNA and encodes a fifth IBV accessory protein, however, in Beaudette the IR contains deletions rendering the sequence more likely a non-coding region [42]. The successful rescue of infectious rIBVs was confirmed by formation of IBV-induced plaques on CK cells (Figure 6A), which were compared to the plaques generated by wild type Beau-R and control virus BeauRΔIR. In contrast to the Δ5ab rIBVs, deletion of the Beaudette IR did not result in smaller plaque sizes when compared to Beau-R (Figure 6A). However, plaque size differences were observed depending on whether the IR sequence was replaced by eGFP or hRluc. Plaques produced from BeauR-eGFPΔIR were approximately 1.5 mm smaller in diameter than those observed from BeauR-hRlucΔIR (Figure 6A). Investigation of the replication kinetics of the rIBVs revealed lower peak titres at 24 hpi for BeauR-eGFPΔIR of 5×10^5^ pfu/ml when compared to peak titres, of approximately 1×10^7^ pfu/ml, for BeauR-hRlucΔIR (Figure 6B); a result consistent with the plaque sizes observed for these viruses. Both Beau-R and BeauRΔIR had similar replication kinetics, showing maximal peak titres of 5×10^7^ pfu/ml at 24 hpi and supporting the plaque assay data; indicating that removal of the IR sequence *per se* did not have any effect on the growth and replication of rIBV BeauRΔIR.

Pursuing the result of the initial observed differences between the replication kinetics of the rIBVs BeauR-eGFPΔIR and BeauR-hRlucΔIR, the replication kinetics of rIBV BeauR-eGFPΔIR R2 were also investigated and found to be identical to those of rIBV BeauR-eGFPΔIR R1 (Figure 6C). This indicated that the replication differences observed between BeauR-eGFPΔIR and BeauR-hRlucΔIR resulted from the insertion of different reporter genes, and suggested that, in terms of virus replication, eGFP was not as well tolerated as hRluc at this genome position. While no differences in replication kinetics were observed for BeauR-eGFPΔIR R1 and R2, sequence analysis of P4 stocks of all ΔIR rIBVs revealed that BeauR-eGFPΔIR R1 did contain an additional change; a double mutation of ^25770^U→A and ^25771^U→A leading to the introduction of a premature termination codon within ORF 5b, resulting in the truncation of the 5b protein from 82 to 29 amino acids. A similar mutation has been previously identified in rIBVs with a Beau-R background [45] however, indicating that this particular mutation is not associated with the presence of heterologous genes.

**Figure 7:**
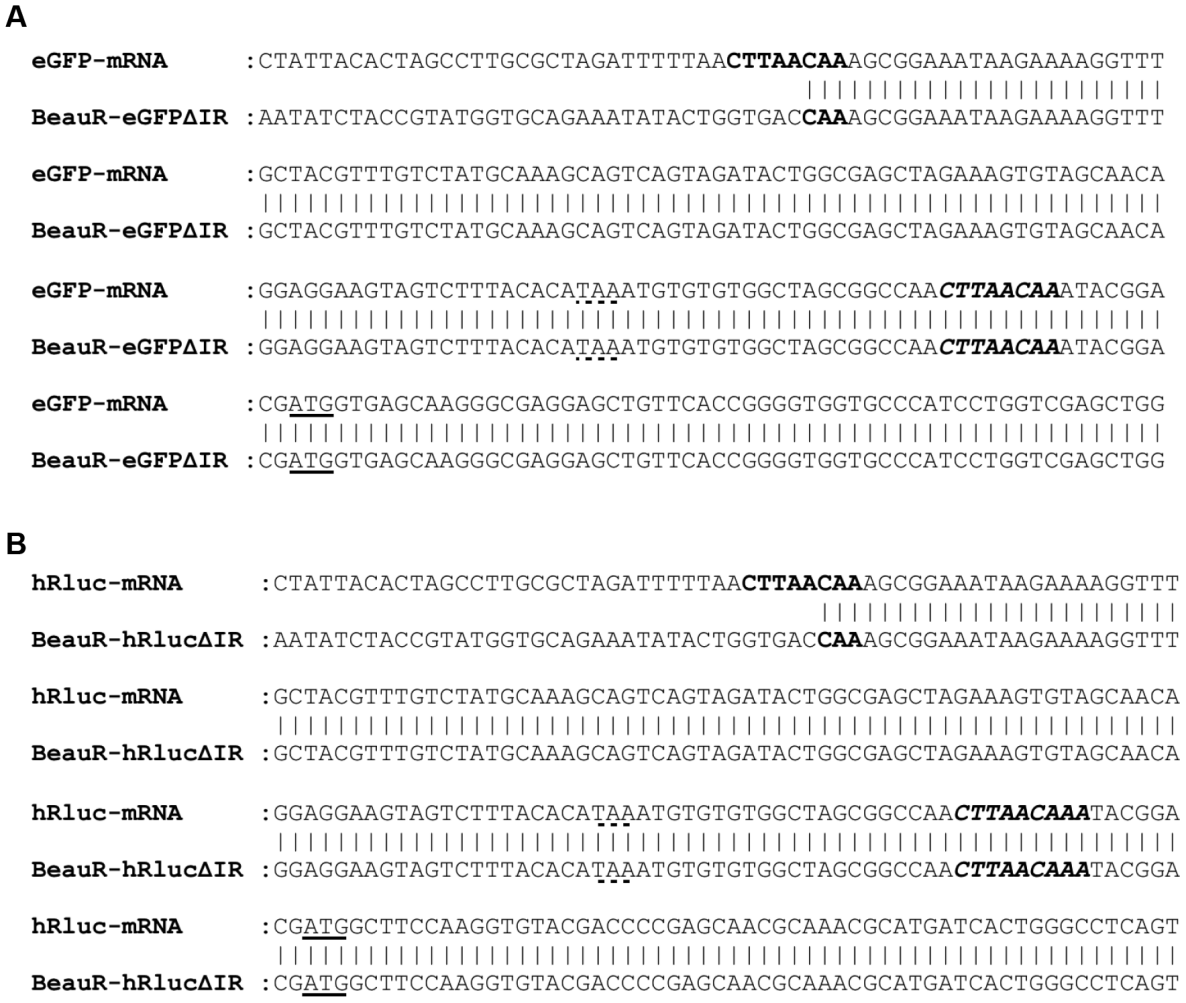
Sequence analysis of sg mRNAs expressing the reporter genes EGFP and hRluc from rIBVs BeauR-eGFPΔIR and BeauR-hRlucΔIR. Total RNA was extracted from CK cells infected with (A) BeauR-eGFPΔIR or (B) BeauR-hRlucΔIR and the reporter gene sg mRNAs amplified by RT-PCR. The sg mRNAs corresponding to eGFP and hRluc were aligned with the reporter gene sequences in the rIBV genomes to identify the origin of the leader-body junctions (in bold). Sequence analysis shows that the sg mRNAs originate from the natural IR TRS and not from the inserted copy of the canonical IBV TRS (in bold, italics). Genomic sequence is shown below and sg mRNA sequence above. The M gene termination codon is shown with a dashed underline and the eGFP (A) or hRluc (B) initiation codon is underlined.

Construction of rIBVs BeauR-eGFPΔIR and BeauR-hRlucΔIR required the insertion of a copy of the consensus Beaudette TRS, CUUAACAA, upstream of the reporter gene sequence for the transcription of an additional sg mRNA from which eGFP or hRluc proteins could be translated. However, northern blot analysis (Figure 6D) revealed that no novel sg mRNAs were generated from the introduced TRS positioned between the M gene and Gene 5. Instead, the non-canonically transcribed sg mRNA recently confirmed for the IR [42] was increased in size as would be predicted for the presence of the reporter gene sequences. Investigation of the larger IR sg mRNA sequences confirmed that they were transcribed via the naturally occurring non-canonical TRS for the IR sg mRNA, identified at nucleotide position 25075 within the M gene of the Beaudette sequence by Bentley *et al*. [42], and not via the artificial copy of the IBV consensus TRS (Figure 7).

Western blot analysis of proteins in rIBV-infected CK cell lysates demonstrated the presence of either eGFP or hRluc (Figure 6E), thus confirming that translation of eGFP or hRluc was not affected by the unexpected observation that the proteins were produced from an existing sg mRNA, rather than from a new sg mRNA generated from an additionally inserted TRS. However, because of the naturally low levels of the IR sg mRNA due to transcription from a non-canonical TRS [42], we consequently observed lower levels of fluorescence from BeauR-eGFPΔIR than from BeauR-eGFPΔ5ab (Figure 4A). Reduced protein expression levels were confirmed by the observation that *Renilla* luciferase activity from BeauR-hRlucΔIR was also lower in comparison to that observed from BeauR-hRlucΔ5ab, with peak activity at 24 hpi approximately 2-log_10_ lower (Figure 4B). It is possible that these reduced protein expression levels may also be a result of reduced translation due to the presence of the wild type IR AUG upstream of the reporter gene AUG; a consequence of the cloning stages of the reverse genetics. This would need to be investigated further to determine the exact cause of the protein levels observed.

### Characterisation of rIBVs expressing hRluc following replacement of ORFs 3a and 3b of Gene 3

**Figure 8:**
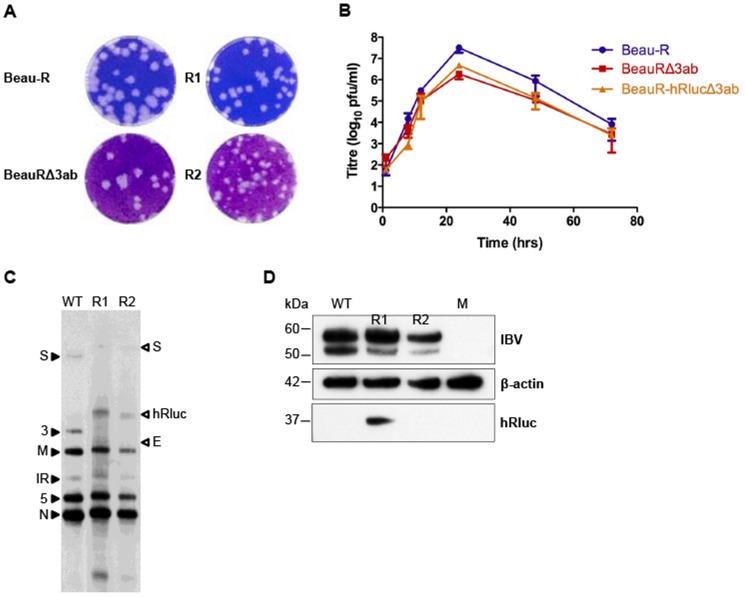
Characterisation of rIBVs with replacement of ORFs 3a and 3b with hRluc. (A) Monolayers of CK cells were infected with wild type virus (Beau-R), control virus (BeauRΔ3ab) or P4 of rIBV BeauR-hRlucΔ3ab and cytopathic effect analysed by plaque assay. (B) Growth kinetics of Beau-R, BeauRΔ3ab and R1 of BeauR-hRlucΔ3ab in CK cells. Extracellular virus was harvested at various times post infection and the progeny virus titres determined by plaque assay. Error bars represent SD of results from three experiments assayed in triplicate. (C) Northern blot analysis of purified mRNA extracted from CK cells infected with 1×10^5^ pfu wild type, parental virus, Beau-R (WT) and replicates R1 and R2 of P4 BeauR-hRlucΔ3ab. Purified mRNA was not quantified prior to loading on gel and is shown only to demonstrate that sg mRNAs were generated as expected. RNAs were hybridised with a DNA probe specific to the IBV 3′ UTR. Closed arrowheads indicate the sizes of sg mRNAs from Beau-R and open arrowheads indicating the altered sizes for the sg mRNAs produced from the rIBVs. The new sg mRNA (labelled E), synthesised from the inserted TRS for expression of the E protein, is indicated with an open arrowhead. (E) Western blot analysis of reporter gene expression. Proteins in cell lysates of CK cells infected with wild type, parental virus, Beau-R (WT) or P4 BeauR-hRlucΔ3ab R1 and R2 were analysed by western blot with anti-IBV, anti-hRluc and anti-actin antibodies. Cell lysates from uninfected cells (M) were included as a negative control.

Using reverse genetics we were able to rescue two replicates of rIBV BeauR-hRlucΔ3ab, in which ORFs 3a and 3b were replaced by hRluc. For generation of rIBV BeauR-eGFPΔ3ab we found that although the initial stages of the reverse genetics were successful, infectious rIBV could not be rescued by passage on CK cells, or in embryonated hens' eggs. The successful rescue of two replicates of rIBV BeauR-hRlucΔ3ab was demonstrated by the formation of IBV-induced plaques in CK cell culture (Figure 8A). While similar sized plaques had been observed between replicates of the Δ5ab and ΔIR recombinants this was not the case for BeauR-hRlucΔ3ab, where a mixture of plaque sizes was visible for R2. Such a variation in plaque size is often an indication of a mixed population and the observed plaque phenotype suggested that BeauR-hRlucΔ3ab R2 was not rescued as a stable molecular clone. This was confirmed by sequence analysis which identified sequence variation in a region initiating approximately 40 nts from the 3′ end of the hRluc gene in R2 and extending into the inserted TRS required for the transcription of a sg mRNA for the E protein (data not shown). Additionally, a 3 nt deletion was also observed within the inserted canonical, CUUAACAA, TRS for the E protein in R1 of BeauR-hRlucΔ3ab, resulting in a non-canonical TRS sequence of CUUAA. The presence of nucleotide changes and deletions in this region suggested that the stability of these viruses was not optimal and that the introduced sequences required to ensure transcription of the genes were not well tolerated. These observations may offer an explanation for the failure of rIBV BeauR-eGFPΔ3ab to rescue as fatal deletions may have arisen in this region during the initial stages of rescue and/or passage.

The replication kinetics of rIBV BeauR-hRlucΔ3ab R1 were investigated and demonstrated peak titres at 24 hpi that were similar to control virus BeauRΔ3ab with both approximately 1-log_10_ lower than wild type Beau-R virus (Figure 8B). It should be noted that transcription of the sg mRNA for expression of the E gene from BeauRΔ3ab was from the normal Gene 3 TRS [41]. Heterologous gene expression was confirmed by northern blot analysis (Figure 8C) with an expected increase in size of the Gene 3 sg mRNA observed when compared to parental virus Beau-R. However, the size of the hRluc sg mRNA of R2 appeared smaller than that of R1 suggesting that the issues identified above, and confirmed by sequence analysis, may be due to deletions within the hRluc gene. Analysis of IBV RNA from CK cells infected with BeauR-hRlucΔ3ab R1 identified a low abundant sg mRNA, at the expected position above the M gene sg mRNA, representing the sg mRNA produced from the inserted TRS for the expression of the E protein. The low abundance of this new sg mRNA could result from an effect of the observed deletion in the introduced TRS, and/or may result from the fact that synthesis of a sg mRNA produced from an introduced TRS may not be as efficient as that for normal viral sg mRNA transcription. Introduction of a new TRS within the IR did not result in synthesis of a new mRNA species.

Western blot analysis identified that the hRluc protein was expressed from BeauR-hRlucΔ3ab R1 in infected CK cells but not from R2 (Figure 8D). This confirmed that the sequence mutations observed within the hRluc gene of BeauR-hRlucΔ3ab R2 affected the expression of hRluc protein, and also potentially prevented transcription of a sg mRNA for the E protein. Luciferase activity was detected in cell lysates from cells infected with R1 (Figure 4B) and peak levels at 24 hpi were found to be similar to those produced by rIBV BeauR-hRlucΔIR. Characterisation of R1 and R2 of BeauR-hRlucΔ3ab demonstrated that rescue of rIBVs may result in differences arising between replicates of the same virus.

### Stability of rIBVs expressing heterologous proteins

**Figure 9:**
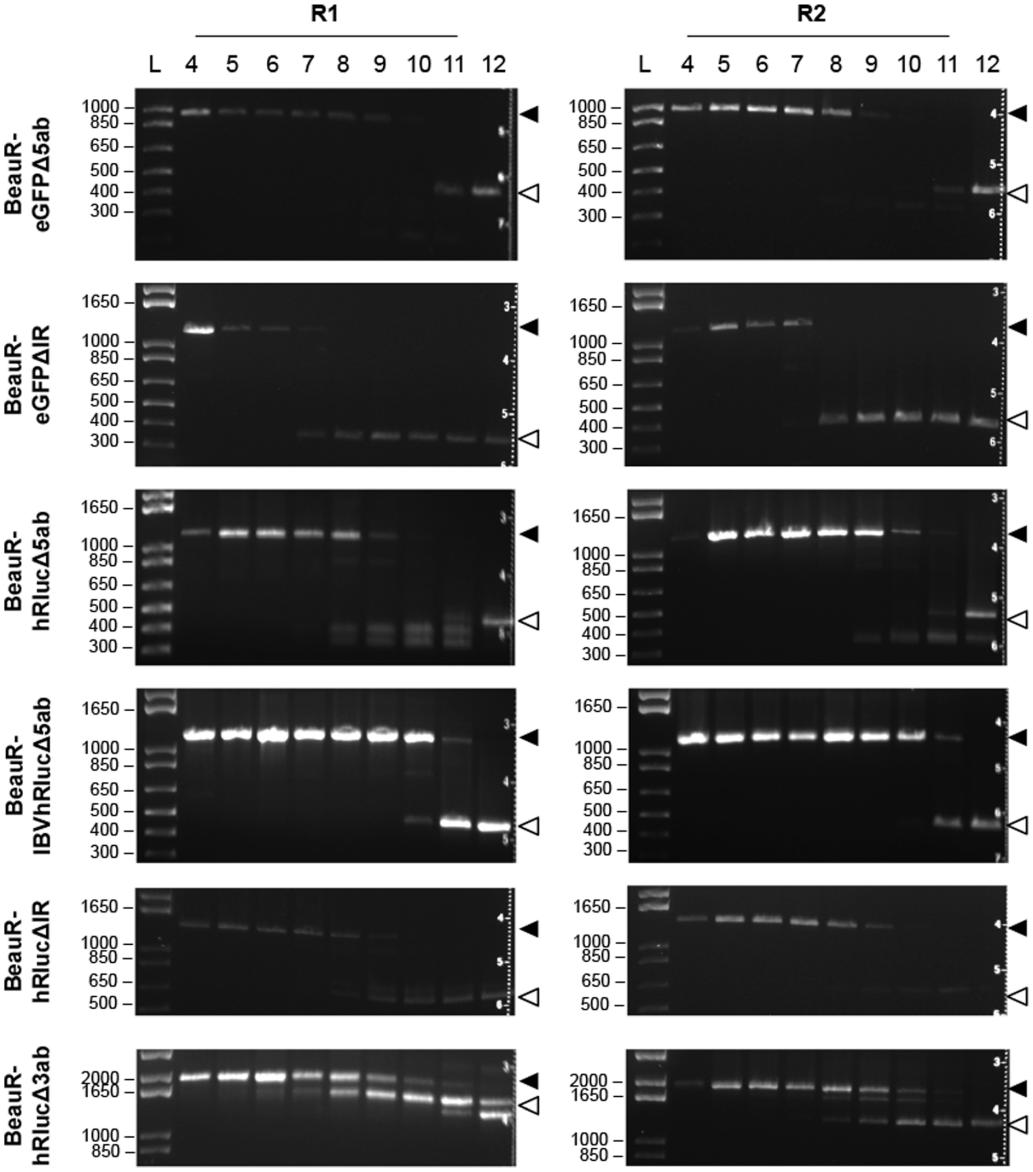
Analysis of genetic stability of the rIBVs. Replicates R1 and R2 of BeauR-eGFPΔ5ab, BeauR-eGFPΔIR, BeauR-hRlucΔ5ab, BeauR-IBVhRlucΔ5ab, BeauR-hRlucΔIR and BeauR-hRlucΔ3ab were blind passaged on CK cells from P4 to P12. Extracellular virus was harvested 24 hpi and RNA analysed by RT-PCR in which the virus genomes were amplified using primers flanking the inserted reporter genes (Table 2) and visualised by gel electrophoresis. Observation of expected PCR products representing genetically stable virus is indicated to the right by closed arrowheads. Genetic instability was attributed to the appearance of smaller PCR products, identified by open arrowheads, representing deletions within the reporter gene sequences. Size markers (L) in base pairs are indicated to the left.

**Figure 10:**
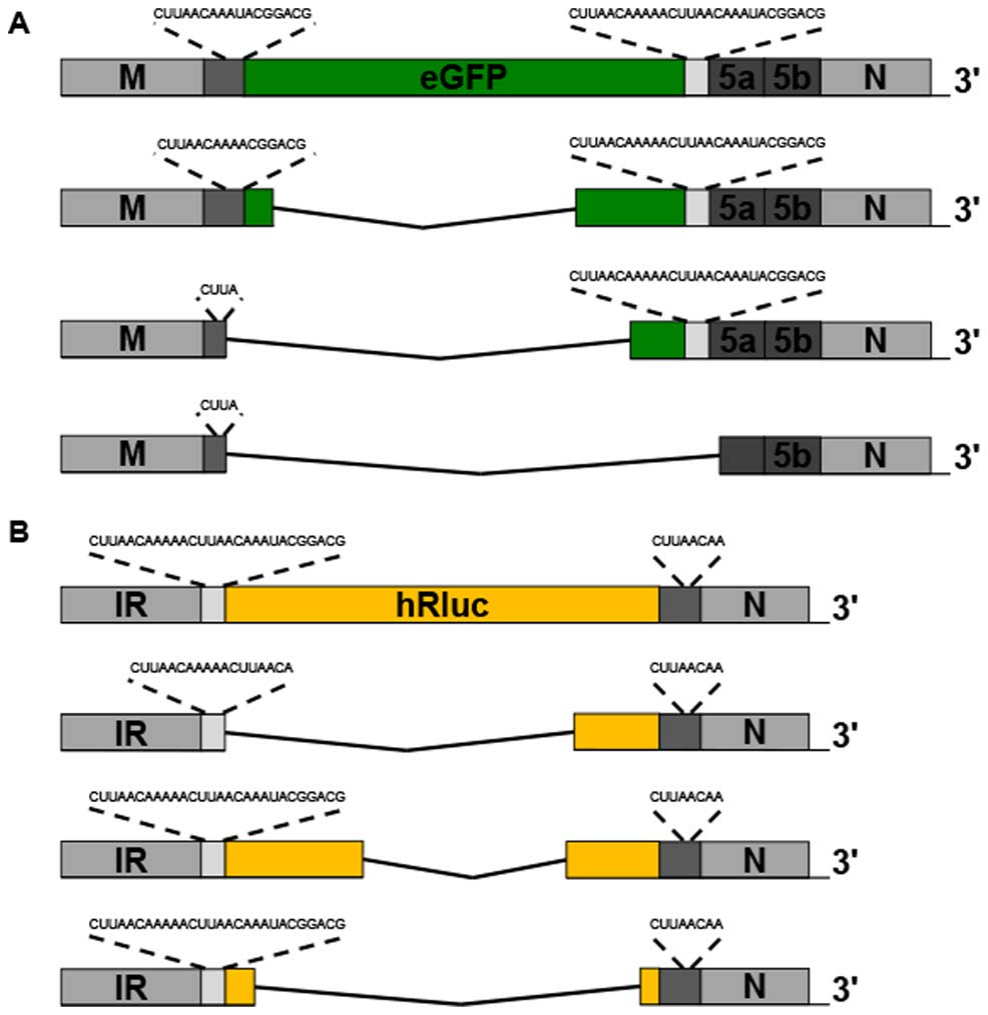
Schematic representation of rIBV genetic instability. Regions flanking the reporter gene sites of rIBVs following passage to P8 on CK cells were amplified by RT-PCR, non-wild type PCR products were cloned and sequenced. Sequence analysis of independently selected clones identified that the RT-PCR products contained a variety of deleted sequences when compared to the sequences of the reporter gene containing IBV cDNAs used for generating the rIBVs. Schematics representing deletions are shown for wild type (A) BeauR-eGFPΔIR and (B) BeauR-hRlucΔ5ab with three example deletions of each highlighting loss of eGFP or hRluc gene sequences. Relevant TRS sequences are shown expanded above each genome.

In order to further assess the potential for using rIBVs as vaccine vectors it was necessary to analyse the stability of each rIBV with respect to the ability to maintain expression of the heterologous gene. The genetic stability of each rIBV was initially assessed by blind passage of the rIBVs on CK cells and characterisation by RT-PCR analysis to determine the presence, or absence, of the heterologous gene (Table 2; Figure 9). As summarized in Table 3, we observed instability arising at different passage numbers, depending on the rIBV, as shown by the gradual loss of the PCR product representing the heterologous gene, and the appearance of smaller PCR products indicating deletion of the heterologous gene sequences (Figure 9). Passaging of rIBVs in embryonated hens' eggs revealed the same pattern of genetic instability for each virus highlighting that selection pressures were the same *in vitro* and *in ovo* (data not shown). An investigation of these smaller PCR products demonstrated that each one represented a different deletion corresponding to the inserted heterologous gene sequence, as shown by examples in Figure 10. Sequence analysis of the smallest PCR product generated during passage of BeauR-eGFPΔIR demonstrated that deletions were not limited to the heterologous gene, and could expand into flanking regions of accessory genes (Figure 10A) as also demonstrated in a study by Shen *et al*. [24].

**Table 2:** Genetic Stability PCR Primers and Expected Product Sizes.

Virus	Primer Sequences^a^	Genome Location^b^	PCR Product (bp)
BeauR-eGFPΔ5ab	F: GAGCTATTAACGGTGTTACC	25306–25325	963
BeauR-hRlucΔ5ab	R: AATCTAATCCTTCTCTCAGA	25741–25760	1179
BeauR-IBVhRlucΔ5ab			1179
BeauR-eGFPΔIR	F: GAATGGTGTTCTTTATTG	24945–24962	1087
BeauR-hRlucΔIR	R: TCTAACACTCTAAGTTGAG	25549–25567	1303
BeauR-hRlucΔ3ab	F: TGACGAATTGTCAAAATG	23457–23474	1916
	R: AGACAGACACGCAAACACTG	24761–24780	

a F  =  forward and R  =  reverse primer.

b As derived from the sequence of the wild type Beau-R strain.

**Table 3:** Genetic Stability of rIBVs.

Virus	Detection of instability^a^	
	R1	R2
BeauR-eGFPΔ5ab	P9	P8
BeauR-eGFPΔIR	P7	P7
BeauR-hRlucΔ5ab	P8	P9
BeauR-IBVhRlucΔ5ab	P10	P10
BeauR-hRlucΔIR	P8	P8
BeauR-hRlucΔ3ab	P5	P7

a Final passage number achieved including rescue passages, P0–P4

Comparison of the stability of the rIBVs showed similar results for each reporter gene, with the loss of eGFP detectable at P7 when replacing the IR and at P8/P9 when replacing Gene 5. The loss of hRluc was detectable at P8 when replacing the IR and also at P8/P9 when replacing Gene 5. Codon-optimisation of the hRluc gene in BeauR-IBVhRlucΔ5ab resulted in an increase in stability by 1–2 passages. Sequence analysis of BeauR-hRlucΔ3ab identified that by P4 errors had arisen in the region of the hRluc gene, which were responsible for the loss of hRluc expression from R2. Despite this, RT-PCR analysis indicated that large-scale deletions, as evidenced by smaller PCR products, did not arise until P7. In comparison, smaller PCR products representing possible deletions were observed as early as P5 for BeauR-hRlucΔ3ab R1, which may have resulted from the 3 nt deletion observed at P4 within the inserted TRS for expression of the E gene. Overall, analysis of the stability of BeauR-hRlucΔ3ab identified that passaging of this rIBV led to the rapid accumulation of sequence errors and subsequently deletion of the heterologous gene sequence.

**Figure 11:**
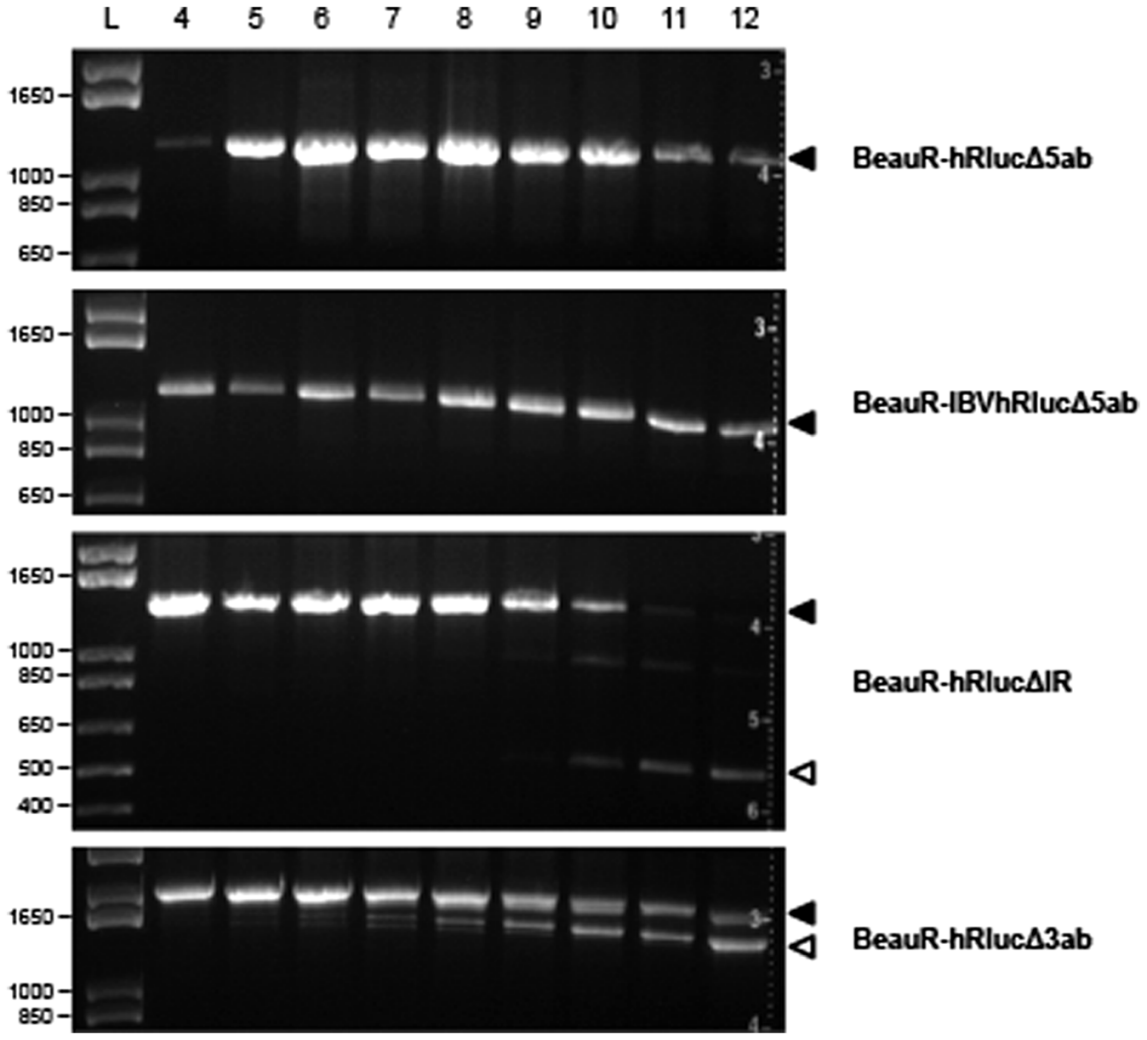
Analysis of the genetic stability of the rIBVs following passage at a low MOI. Replicates R1 of BeauR-hRlucΔ5ab, BeauR-IBVhRlucΔ5ab, BeauR-hRlucΔIR and BeauR-hRlucΔ3ab were passaged on CK cells from P4 to P12 at an MOI of 0.01. Extracellular virus was harvested 24 hpi and RNA analysed by RT-PCR in which the virus genomes were amplified using primers flanking the inserted reporter genes (Table 2) and visualised by gel electrophoresis. Observation of expected PCR products representing genetically stable virus is indicated to the right by closed arrowheads. Genetic instability was observed for BeauR-hRlucΔIR and BeauR-hRlucΔ3ab as indicated by the appearance of smaller PCR products (open arrowheads) and represented deletions within reporter gene sequences. Size markers (L) in base pairs are indicated to the left.

In this study we also compared the genetic stability of rIBVs following passaging at a low MOI of 0.01 to prevent the potential accumulation of D-RNAs, a known consequence of passaging IBVs in cell culture [43], [49]. Analysis of the genetic stabilities of R1 from each of the rIBVs expressing hRluc, following passage at an MOI 0.01, identified that genetic stability was maintained longer for rIBVs in which Gene 5 was replaced (Figure 11). Expression of hRluc, and IBVhRluc, was stable for at least 12 passages in cell culture following replacement of Gene 5; an increase of at least 4 passages when compared to passage at a higher MOI. A difference of only one passage number was observed for the replacement of the IR while no increase in stability was observed for replacement of ORF3a and 3b, suggesting the stability of these rIBVs could not be improved. Overall these results indicated that the replacement of Gene 5 resulted in rIBVs with the highest genetic stability and that this stability could be improved by controlling the MOI at which virus is passaged.

## Discussion

In this study we have confirmed that heterologous gene sequences can be inserted into the IBV Beaudette genome, resulting in the expression of non-IBV proteins and therefore supporting the potential development of IBV-based vaccine vectors. Infectious rIBVs were obtained with the reporter gene hRluc replacing the nonessential genes Gene 5, IR and ORFs 3a and 3b of Gene 3, and with the reporter gene eGFP replacing Gene 5 and the IR. An additional rIBV was obtained in which Gene 5 was replaced with an IBV codon-optimised version of the hRluc gene, IBVhRluc. The genetic stability of rIBVs expressing heterologous genes was primarily found to be dependent on the genomic location at which heterologous genes were incorporated with the replacement of Gene 5 yielding the most stable recombinants; stable expression of wild type, and codon-optimised, hRluc was achieved for at least 12 passages *in vitro* when passaged at an MOI of 0.01.

Coronaviruses have several attributes that favour them as an attractive vehicle for development as vaccine vectors. Coronaviruses possess large genomes and, in all cases, accessory genes have been identified that are not essential for virus replication and can therefore be deleted, thus providing suitable targets for the insertion of heterologous genes [27], [40], [41], [50]–[52]. The replacement of accessory genes with heterologous genes has been successfully demonstrated for a number of coronaviruses in recent years, including IBV, and has often been shown to result in more genetically stable recombinant viruses when compared to expression of heterologous genes from novel sg mRNAs [23]–[27]. For this reason we chose to focus on rIBV constructs in which heterologous genes were used to replace existing accessory genes and demonstrated that, in the absence of deletions within the reporter gene sequence, such as observed for BeauR-hRlucΔ3ab R2, heterologous protein expression was possible from three different regions of the IBV genome: Gene 5, the IR and ORFs 3a and 3b (Figures 3D, 6E and 8D).

The location of a heterologous gene within a coronavirus genome was shown to be one of the determining factors in the genetic stability of recombinant coronaviruses [29] and was subsequently confirmed in this study. We have demonstrated that the genetic stability of the rIBVs correlated with the location of a reporter gene, which indirectly correlated with the level of sequence modifications required in order to accommodate the reporter gene in the IBV genome. The replacement of Gene 5 with hRluc or IBVhRluc yielded rIBVs that were stable to at least P12 when passaged at an MOI of 0.01 on CK cells (Figure 11). When Gene 5 was replaced with eGFP rIBVs were stable to P8/P9 in initial experiments; a similar level of stability was observed by Youn *et al*. [27] in which a rIBV expressing eGFP from replacement of ORF 5a alone was stable to P5 in Vero cells. In our initial experiments no difference in stability was observed between rIBVs expressing heterologous genes from Gene 5 or from the IR with all viruses stable to between P7 and P9 (Figure 9). However, in contrast to the replacement of Gene 5 where stability was increased by at least 4 passages, no difference was observed in the stability of BeauR-hRlucΔIR when passaged at an MOI of 0.01. This suggests that controlling the MOI at which viruses are passaged may only be beneficial for some rIBV constructs; in this case the replacement of Gene 5.

In contrast to Shen *et al*., who obtained a rIBV, genetically stable up to P15, by replacing ORFs 3a and 3b with firefly luciferase [24], we observed that insertion of heterologous sequences in this position resulted in viruses arising rapidly with deletions within the hRluc gene; appearing from P5 onwards (Figures 9 and 11). This variation in observed stability may be accounted for by the intrinsic differences between the luciferase genes. However, a previous study identified that in the case of MHV the firefly luciferase was less stable than hRluc when inserted at the same genome position [28]. In both this study and that of Shen *et al*. expression of heterologous genes following replacement of IBV ORFs 3a and 3b required further modification to the virus sequences to ensure the continued transcription of ORF 3c, the E protein. The contradictory observations regarding the expression of heterologous genes from IBV Gene 3 suggest the possibility that not only the position of the heterologous gene is important for stability, but also the way in which genome sequences are modified to accommodate the heterologous gene.

Interestingly, for BeauR-hRlucΔ3ab, despite deletions arising rapidly in the population from P5 onwards, we observed the expression of hRluc up to P11 in initial experiments and up to at least P12 when passaged at an MOI of 0.01 (data not shown). This was in contrast to the replacement of Gene 5 or the IR, where once deletions were detected loss of the heterologous gene from the virus population was complete following 1–3 further passages. Although the replacement of ORFs 3a and 3b led to rapid accumulation of deletion variants within the virus population it took longer for these variants to become the dominant population compared to the replacement of Gene 5 or the IR. These observations highlight the requirement to analyse stability by sequence analysis rather than expression of the heterologous protein alone. Overall, our results raise the suggestion that the replacement of ORFs 3a and 3b may be a suitable target if the sequence of the construct can be designed to prevent errors arising around the inserted TRS for expression of the E protein that undoubtedly led to the rapid expansion of deletions within this region.

In order to accommodate the expression of heterologous genes via novel sg mRNAs artificially inserted TRSs are also required upstream of the heterologous gene. In this study two such TRSs were required: (1) for expression of E from BeauR-hRlucΔ3ab and (2) for expression of the reporter genes from BeauR-eGFPΔIR and BeauR-hRlucΔIR. As discussed above mutations were encountered within the TRS for E from BeauR-hRlucΔ3ab that likely contributed to the overall instability of the virus in this region. The design of the TRS may have played a role in this through inefficient synthesis of the E sg mRNA, and suggests a sub-optimal TRS design. Similarly, in the ΔIR rIBVs the TRS inserted for expression of eGFP and hRluc may be sub-optimal thus leading to the use of the existing non-canonical TRS-B for the IR. In both cases there was limited complementarity between the artificially inserted consensus TRS and the leader sequence of the virus beyond the CUUAACAA sequence. It is possible that increasing this complementarity would lead to more stable gene expression or, in the case of the IR, the switch in transcription occurring at the artificial TRS and not the natural non-canonical IR TRS. However, as discussed in the design of the artificial TRS's the full extent of the TRS required for IBV sg mRNA transcription has yet to be elucidated and increased knowledge in this area would be required to better design recombinant viruses expressing heterologous genes as additional transcripts.

Our results confirmed those from other studies that the cause of virus instability, with respect to expression of the heterologous gene, resulted from deletion of the heterologous gene sequences [24], [25], [27], [28]. The proposed mechanism for this is via homologous, or non-homologous, recombination events between sequences of virus genomes, such as TRSs that would result in the loss of the heterologous gene and it is probably also the situation with the rIBVs analysed in this study. Following analysis of the types of deletions arising in the virus populations we observed that the majority of deletions began within, or close to, the TRS associated with transcription of the heterologous gene (Figure 10). As these are regions of high similarity it suggests that they are potential hotspots for homologous recombination events during coronavirus replication. In rIBVs in which the heterologous genes replaced the IR and ORFs 3a and 3b sequences, an additional TRS was inserted into the IBV genome, when compared to wild type sequence or Gene 5 replacement. The presence of an additional TRS would have provided an additional target for recombination events and may in part explain the difference in stability observed for each rIBV construct.

In this study we also sought to examine the effects of codon-optimisation of heterologous genes to establish whether there would be any advantages in designing vaccine vectors with this in mind. Codon-optimisation of genes has been shown to increase the translational efficiency of heterologous genes in different cell types by balancing the tRNA requirements of the gene with those most available in a particular host cell type. Expression of viral genes has also been shown to be increased by codon-optimisation [53], [54] or decreased by codon-deoptimisation [55], and this may be important for expression of heterologous genes from vaccine vectors. Codon usage may also have a bearing on RNA stability by altering the secondary structures of RNAs, and so changes to codon usage may affect the overall stability of viruses expressing heterologous genes. We took the approach of altering every codon of the heterologous genes to those preferentially used by IBV in order to find out if reporter protein expression and/or virus stability would be improved. We found that protein expression of codon optimised hRluc, IBVhRluc R2, was approximately 2-log_10_ lower than standard hRluc from 24 hpi when expressed in CK cells (Figure 4B) demonstrating that in this case translation efficiency was reduced by codon-optimisation. It is possible that codon optimisation has resulted in the introduction of sequence elements that limits the efficiency of protein expression in avian cells, thus counteracting the original optimisation of Rluc by Zhuang et al. for expression in mammalian cells [56]. Our results suggest that codon-optimisation may potentially result in improved virus stability, as a 1 or 2 passage increase in stability was observed for BeauR-IBVhRlucΔ5ab during initial passaging experiments compared to BeauR-hRlucΔ5ab. However, a complete investigation of the translation efficiency of a codon optimised gene, using synthetic RNAs, would be required if such a modification were to be pursued for vaccine development.

Overall our data has shown that a number of rIBVs were capable of expressing a heterologous gene, offering the potential for developing IBV as a vaccine vector. The stability of these rIBVs varied depending on genome location of the heterologous gene and/or the level of modification to the IBV genome required for expression of the gene, or to ensure expression of essential IBV proteins following insertion of the heterologous gene. The replacement of Gene 5 proved to be the most successful in terms of stability while the replacement of ORFs 3a and 3b was found to be the least stable. Overall, using the reporter genes described in this work, the type of heterologous gene inserted was generally found to have little effect on protein expression or stability. However, as not all eGFP-based rIBVs were rescued comparison to the corresponding hRluc-based rIBVs is limited. Whilst we demonstrated that heterologous proteins could be expressed from several genomic locations a more in-depth analysis quantifying sg mRNA, and protein, expression would be required to determine the optimal design of potential rIBV vectors expressing additional viral proteins.

The data presented demonstrates that IBV could be used as a vaccine vector to generate bivalent vaccines capable of protecting chickens against IB as well as a second disease based on expression of heterologous genes replacing Gene 5. The coronavirus spike gene is a known determinant of viral tropism [57] and we have previously demonstrated the ability of swapping spike genes between strains of IBV [46], [57]. This raises the possibility of exchanging the spike gene of rIBVs expressing heterologous genes with that of a different coronavirus in order to alter the cellular tropism of the virus and investigate the potential for utilising IBV as a broader vector for use in non-avian animal species and humans.
